# Supplementary figures and images for: Ruvbl1 silencing affects reproduction of the corn planthopper, Peregrinus maidis
Source: PLoS One. 2024 Dec 30;19(12):e0316352. doi: 10.1371/journal.pone.0316352 (PMC11684562; doi:10.1371/journal.pone.0316352)

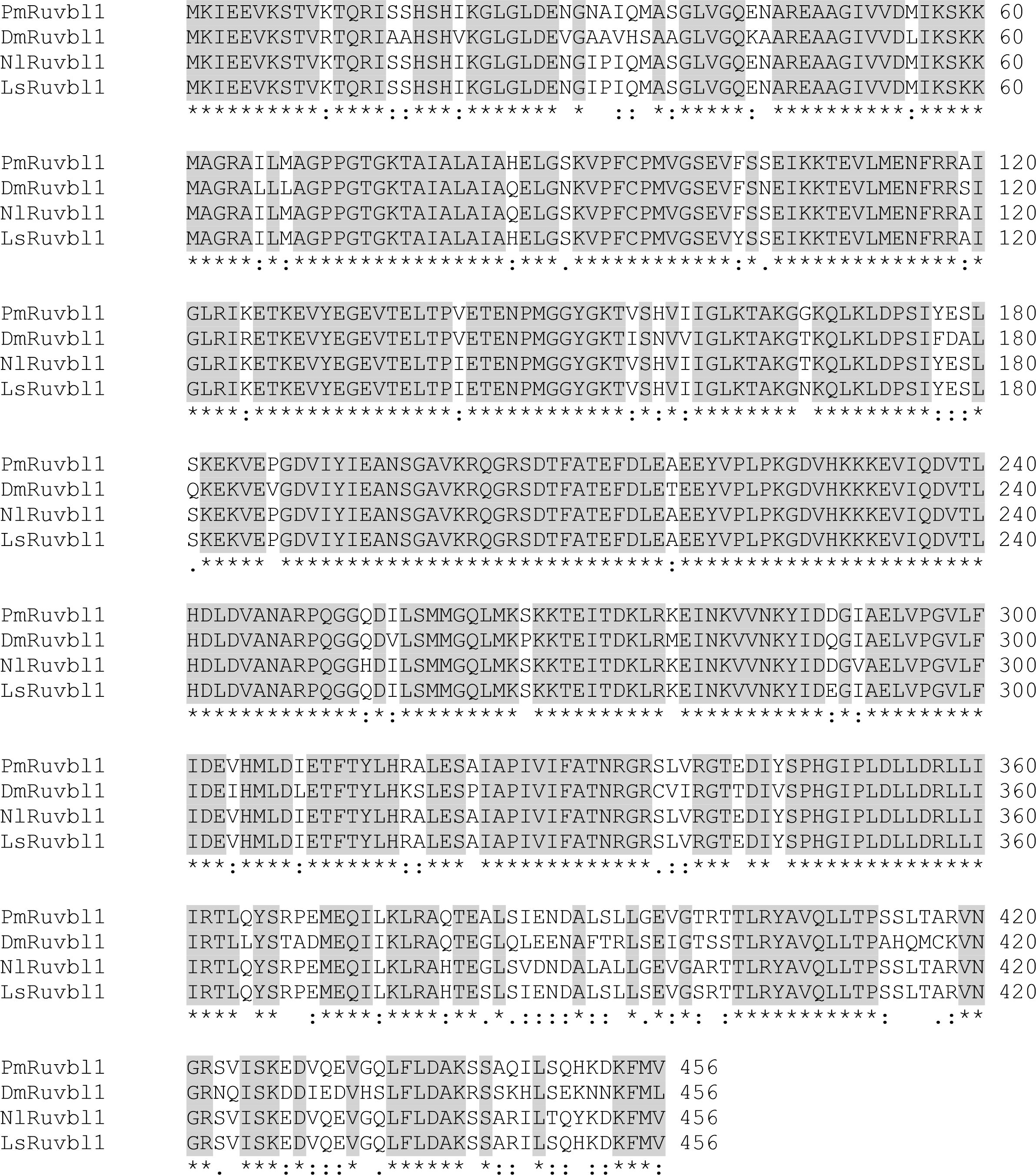

Supplement: S1 Fig — Sequences were aligned using Clustal Omega webserver. Symbols below alignment indicates level of conservation as follows: * identical conserved residues;: strongly similar residues;. weakly similar residues; no symbol indicates residues that are not conserved. Residues highlighted in light gray indicate 100% of conservation. (TIF) [file pone.0316352.s001.tif]

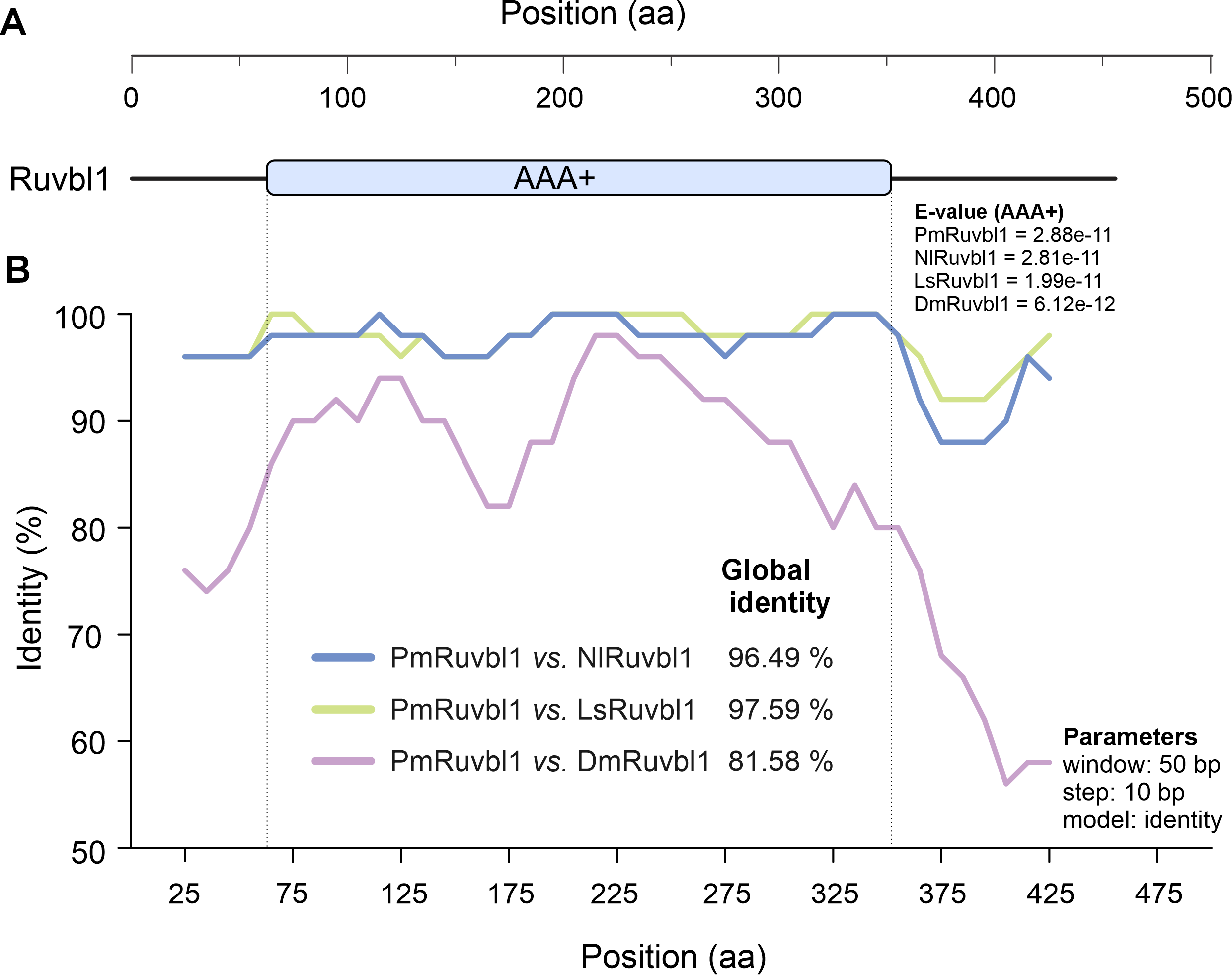

Supplement: S2 Fig — (A) Predicted domain architecture of PmRuvbl1 amino acid sequence using SMART (Simple Modular Architecture Research Tool). The canonical AAA+ superfamily of ATPases associated with a variety of cellular activities are highlighted in light blue and was also predicted for Ruvbl1 from Drosophila melanogaster (Dm), Nilaparvata lugens (Nl) and Laodelphax striatellus (Ls). E-values are shown for each Ruvbl1 (right side) according to SMART prediction. (B) Sliding window pairwise identity of amino acid sequences comparing PmRuvbl1 with those from L. striatellus, N. lugens and D. melanogaster using Simplot++ [32]. Global identity was calculated using Blastp. (TIF) [file pone.0316352.s002.tif]
